# A woman’s worth: an access framework for integrating emergency medicine with maternal health to reduce the burden of maternal mortality in sub-Saharan Africa

**DOI:** 10.1186/s12873-020-0300-z

**Published:** 2020-01-13

**Authors:** Martina Anto-Ocrah, Jeremy Cushman, Mechelle Sanders, Timothy De Ver Dye

**Affiliations:** 1 Department of Emergency Medicine, University of Rochester School of Medicine and Dentistry, Rochester, NY USA; 2 Department of Obstetrics and Gynecology, University of Rochester School of Medicine and Dentistry, 601 Elmwood Ave, Rochester, NY 14642 USA; 3 Division of Pre-Hospital Medicine, Department of Emergency Medicine, University of Rochester School of Medicine and Dentistry, Rochester, NY USA; 4 Division of Health Services Research and Policy, Department of Public Health Sciences, University of Rochester School of Medicine and Dentistry, Rochester, NY USA

**Keywords:** Three delays, Emergency obstetric care, Healthcare access, Africa, Pregnancy, Gender, Maternal mortality, Norms, Community, Sustainable development goals, African Federation of Emergency Medicine (AFEM), World Health Organization (WHO)

## Abstract

**Background:**

Within each of the Sustainable Development Goals (SDGs), the World Health Organization (WHO) has identified key emergency care (EC) interventions that, if implemented effectively, could ensure that the SDG targets are met. The proposed EC intervention for reaching the maternal mortality benchmark calls for “timely access to emergency obstetric care.” This intervention, the WHO estimates, can avert up to 98% of maternal deaths across the African region.

Access, however, is a complicated notion and is part of a larger framework of care delivery that constitutes the approachability of the proposed service, its acceptability by the target user, the perceived availability and accommodating nature of the service, its affordability, and its overall appropriateness.

Without contextualizing each of these aspects of access to healthcare services within communities, utilization and sustainability of any EC intervention-be it ambulances or simple toll-free numbers to dial and activate EMS-will be futile.

**Main text:**

In this article, we propose an access framework that integrates the Three Delays Model in maternal health, with emergency care interventions. Within each of the three critical time points, we provide reasons why intended interventions should be contextualized to the needs of the community. We also propose measurable benchmarks in each of the phases, to evaluate the successes and failures of the proposed EC interventions within the framework. At the center of the framework is the pregnant woman, whose life hangs in a delicate balance in the hands of personal and health system factors that may or may not be within her control.

**Conclusions:**

The targeted SDGs for reducing maternal mortality in sub-Saharan Africa are unlikely to be met without a tailored integration of maternal health service delivery with emergency medicine. Our proposed framework integrates the fields of maternal health with emergency medicine by juxtaposing the three critical phases of emergency obstetric care with various aspects of healthcare access. The framework should be adopted in its entirety, with measureable benchmarks set to track the successes and failures of the various EC intervention programs being developed across the African continent.

## Background

### The maternal mortality burden in the African region, and the role of emergency care services

A pregnant woman in sub-Saharan Africa has the highest risk of dying during childbirth than in any other region of the world [1]. A 15-year-old girl living in this part of the world has a one in 36 risk of mortality during pregnancy and childbirth over the course of her lifetime, compared to a girl the same age residing in Europe whose risk is one in 3300 [1–3].

The majority of maternal deaths cluster around labor, delivery and the 24 h following delivery, and are a consequence of obstetric emergencies, which are life-threatening medical complications that occur any time during pregnancy, labor or birth [4, 5]. Fifteen percent of all pregnancies, regardless of geography, result in obstetric emergencies [6–8]. In most cases, death is avoided with proper antenatal care and labor support (e.g. fetal extraction, caesarean section, or blood transfusions) [6, 9]. In sub-Saharan Africa however, many women die from such complications [9, 10].

The goal of reaching the Sustainable Development Goal (SDGs) [11] of 70 maternal deaths per 100,000 live births by 2030 can only be achieved if adequate emergency care (EC) interventions are put in place to reduce the global burden of obstetric emergencies [11, 12]. Timely *access* to *emergency obstetric care*, the World Health Organization (WHO) estimates, can avert up to 98% of all maternal deaths [5].

### Timely access to obstetric care: examining the *Three-Delays Model*

**Fig. 1:**
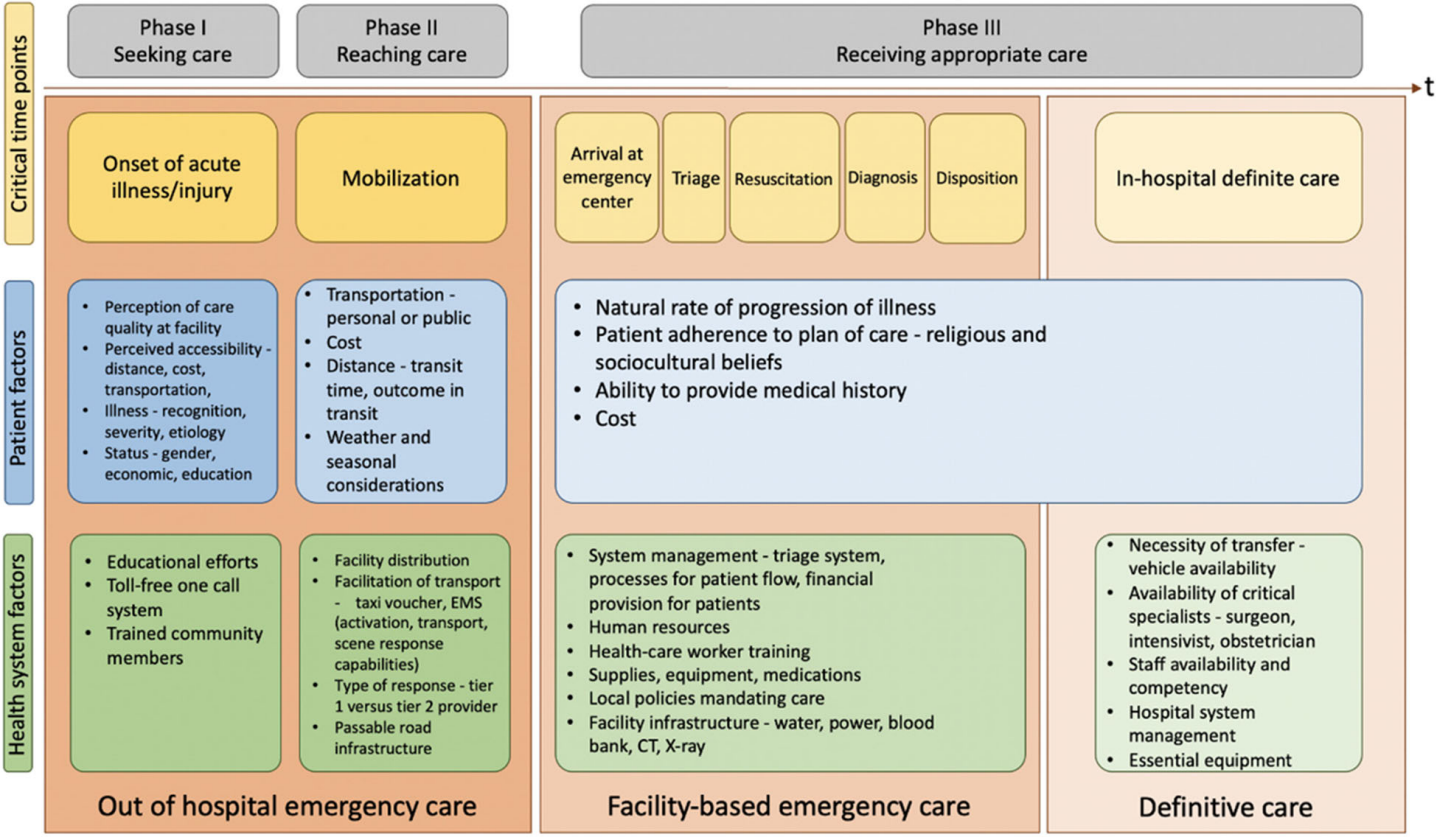
Critical time points in emergency care delivery, framed within the context of the *Three Delays Model* in maternal health. Adapted from Calvello et al [17]

The *Three-Delays Model* [13] has often been used to explain and characterize the factors that contribute to maternal mortality around the world [9, 14–16]. Framed in the context of emergency care (Fig. 1), these factors represent critical delays in (i) the *decision to seek care* during an obstetric emergency (Phase I) (ii) *identifying and reaching* appropriate facilities for care (Phase II), and (iii) *receiving adequate and appropriate care* once the care facility has been reached (Phase III).

**Table 1:** List of Abbreviations, Key Terms and Definitions

Acceptability	One of the five elements of access, defined as the ability to seek healthcare services
Access	A way of approaching, reaching or entering a place, as the right or opportunity to reach, use or visit. Within health care, access is defined as the opportunity or ease with which consumers or communities are able to use appropriate services in proportion to their needs. In this manuscript, defined as having five key elements: approachability, acceptability, availability and accommodation, affordability and appropriateness.
Affordability	One of the five elements of access, defined as the ability to obtain or use the sought-after services
African Federation of Emergency Medicine (AFEM)	The African Federation for Emergency Medicine (AFEM), founded in November 2009, represents a broad coalition of national societies, organisations, and individuals from over 40 countries dedicated to securing high-quality emergency care for all people across Africa.
Approachability	One of the five elements of access, defined as the opportunity to identify healthcare needs
Appropriateness	To actually be offered services that are appropriately suited to the person’s care. One of the five elements of access
Availability and Accommodation	One of the five elements of access, defined as having the desired services available, and being able to reach them
Emergency Care (EC)	The performance of acts or procedures under time sensitive and acute emergency conditions. Often times serves as the first point of contact with the healthcare system for many across the world. May occur in or out of the hospital setting, and may involve physicians, pre-hospital providers and other licensed professionals. Bystanders may also provide emergency care (for cardiopulmonary resuscitation for example), if well trained
Emergency Medical Services (EMS)	A coordinated system of providing emergency care response often in the pre-hospital setting. Includes ambulances, toll-free numbers for dialing emergency services, paramedics and other EMS providers
Emergency Medicine (EM)	The medical specialty concerned with the care of illnesses or injuries requiring immediate medical attention
Maternal Health	Includes the health of women during pregnancy, childbirth, and the postpartum period. It encompasses the health care dimensions of family planning, preconception, prenatal, and postnatal care in order to ensure a positive and fulfilling experience, in most cases, and reduce maternal morbidity and mortality, in other cases
Maternal healthcare delivery	Care delivered to a woman during pregnancy, childbirth, and the postpartum period. Includes antenatal care classes, birth classes, post-partum care etc.
National Ambulance Service (NAS)	Established in the year 2004 as an Agency of the Ghana Ministry of Health, to provide nationwide comprehensive pre-hospital emergency services for Ghanaians. NAS is a product of collaboration between the Ministry of Health and the Ghana National Fire Service of the Ministry of Interior.
Obstetric Emergency	Life threatening medical complications that occur any time during pregnancy, labor or birth
Sustainable Development Goals (SDGs)	a collection of 17 global goals designed to be a “blueprint to achieve a better and more sustainable future for all”. The SDGs, set in 2015 by the United Nations General Assembly and intended to be achieved by the year 2030, are part of UN Resolution 70/1, the 2030 Agenda.
The *Three Delays Model*	A framework for understanding care-seeking behaviors and outcomes in maternal healthcare. Has three phases: Delay in seeking care (Phase I), Delay in reaching care (Phase II) and delay in receiving care (Phase III)
World Health Organization (WHO)	specialized agency of the United Nations that is concerned with international public health. It was established on 7 April 1948, and is headquartered in Geneva, Switzerland.

Though the model is often presented as if the phases are orderly, chronological, and sequential, studies show that this is rarely the case [13, 17–20]. A platitude of interacting factors--both at the patient and health-system levels--add complexities to each of the three critical time points, exacerbating the delay experienced in each phase *exclusively*, and the entire model as a *whole*. These multi-level factors are often complicated by cultural, socio-economic, and infrastructural expectations that oscillate between the three phases, creating a cascade of events that often places the pregnant woman at risk for adverse obstetric outcomes [13].

In Phase I, for example, the decision to seek professional help for a laboring woman (prior to or during the onset of labor) may be influenced by gender and cultural norms of decision making. The woman’s husband, parents, in-laws, household, or community as a whole [10, 13, 14, 16, 19] may be responsible for deciding *when* and *if* she should need trained labor support for delivery. The decision-maker is often the cost bearer, who (given their financial responsibilities) may opt the woman out of delivering at a healthcare facility [10, 13, 14, 16], in favor of delivering locally and potentially risking her life. Even if it is decided a priori that the women will deliver at a health care facility, reaching that facility, or transferring patients between facilities (Phase II) may be impossible, given infrastructural barriers such as poor roads, availability of transport and geography [10, 13, 21, 22]. The types of care the woman needs once she arrives at the facility (Phase III) are governed not only by the severity of her situation, but also by institutional policies of staffing, training, and types of equipment [1, 14, 15, 20, 23] available at the facility.

In this third phase of the model, the quality of care a woman receives during her labor experience --and the results of that care-- will consequentially affect future decision-making processes (in Phase I) [13, 24], making the seemingly linear *Three Delays Model* cyclical-by default.

### Situating the *Three Delays* within the context of emergency care delivery in the African region

Two of the phases of the *Three Delays Model* (Phases I and II, Fig. 1) occur in the out-of-hospital setting where Emergency Medical Services (EMS), a coordinated system of EC response [25], have been identified by the WHO as an effective public health intervention for addressing the disproportionately high burden of maternal mortality [26–35]. Across the African region, pre-hospital emergency care systems are being developed to provide care and transportation for critical, time-sensitive obstetric and other emergencies for diverse populations of varying socioeconomic strata [26, 28–39]. Approximately 98 million Africans in 16 countries are geographically served by an EMS agency or system [40]. Countries like South Africa, North Sudan, Nigeria, Ethiopia, and Ghana have some of the continent’s most advanced EMS systems [29, 40]; whereas others like the West African country of Sierra Leone, which bears one of the region’s highest maternal mortality rates, are just getting their EC programs off the ground [41, 42].

The *availability* of EMS and other essential components of an EC system however, *does not guarantee access* to, or utilization of those services. Emerging findings from Ghana support this phenomenon. Ghana boasts one of sub-Saharan Africa’s thriving EMS systems. Formed in 2004, the National Ambulance System (NAS) comprises a fully operational ambulance fleet with 199 basic life support-equipped ambulances and 1651 emergency medical technicians (EMTs) [29, 35]. The NAS covers 81% of the Ghanaian territory, and provides emergency services for the 26million Ghanaian citizens [29, 35]. Similar to the US’ 9–1-1 EMS activation system, NAS services are activated when dialers call 1–9-3 [29]. Despite these EMS activation systems in place, population studies [29, 43] show consistently that ambulances are the least preferred modes of emergency transport for Ghanaian study participants. Survey respondents deem ambulances as slow, unreliable, and best suited for transporting corpses [29, 43]. Consistently, the majority of Ghanaians surveyed indicate that they would prefer taxis and commercial vehicles for emergency transport, over the NAS’ services.

These findings give emphasis to the notion that *availability does not equal access*. The *availability* of EMS, ambulances, toll free-numbers and the various aspects of EC across various parts of sub-Saharan African, *may not translate to utilization,* if contextually appropriate systems are not put in place to allow targeted communities to properly *access* the implemented resources [44, 45]. For EC to be fully delivered, and emergency medicine fully adopted, implementation scientists must understand the complexities of *access;* particularly within the African context.

**Fig. 2:**
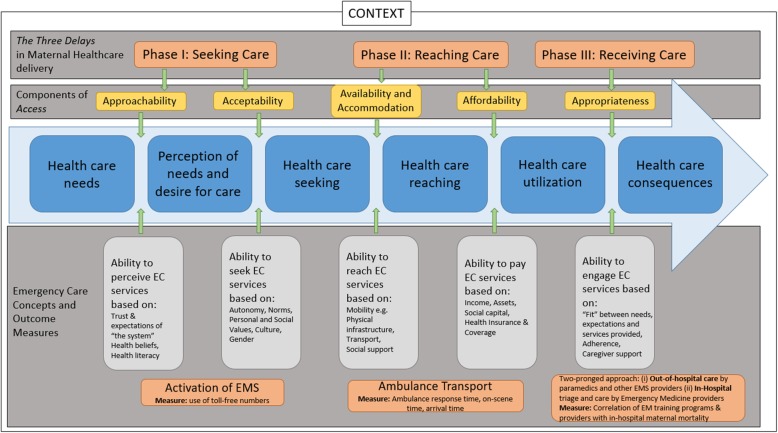
Conceptual Framework of Access to Emergency Care Services within the Context of the *Three Delays Model.* Adapted from Levesque et al [44]

Thus in this article, we propose a conceptual framework that integrates the five aspects of access [44] with the *Three Delays Model* in maternal health and emergency medicine (Fig. 2). The model offers opportunities for implementation scientists to contextualize EC interventions to the specific needs of the community in which the intervention programs are implemented, and institute measurable, attainable and realistic benchmarks to track the successes and failures of the intended interventions.

## Main text

### “Access”, the *Three Delays* and emergency care delivery

*Access* (Fig. 2) is a complex construct, that incorporates five key elements [44]: the opportunity to identify healthcare needs (approachability), to seek healthcare services (acceptability), to reach the healthcare resources (availability and accommodation), to obtain or use the sought-after services (affordability), and to actually be offered services that are appropriately suited to the person’s care (appropriateness). Thus *access* has elements of supply and demand, and is determined by multi-level, patient, and infrastructural factors that either enable the notion of access, or hinder it.

### The first delay (phase I): seeking care-approachability and acceptability

#### Access concepts

Within access, approachability (the ability to perceive that EC services are required) and acceptability (the ability to seek EC services) are part of the care-seeking attributes of the first delay (Phase I of the *Three Delays Model*) in maternal health theory. The *individual has to subjectively decide that they require emergency care and treatment* based on a set of personal health beliefs, their health literacy, trust, and expectations of the healthcare system. However, their ability to seek the desired services depends on* if they have the personal  autonomy* to seek the care they perceive they need. Autonomy is determined by a set of norms and expectations that are attributed to individuals in a given society. For example, is it culturally appropriate for a woman to solely decide to seek EC services, or is that decision up to her spouse, in-laws or community to make? Is the woman an autonomous decision maker of her own fate?

#### Emergency medicine benchmarks

In this phase of the integrated framework, depicted in Fig. 2, implementation scientists should focus on “EMS normalization”. The community should be educated about EMS operations and the life-sustaining benefits of using ambulance services over taxis and other traditional modes of transport. Education curricula should cover how ambulances are alerted (toll free numbers), qualifications and training of EMS providers, types of life-sustaining care for maternal and other emergencies provided on ambulances versus commercial vehicles, etc. Barriers to approachability and acceptability will be unique to each community, based on their distinct social structures and cultural beliefs. Thus interventions for “EMS normalization” through education for example, have to be tailored to the community’s norms. Using a “one size fits all” approach to implementation is doomed to fail from the outset.

A measurable outcome in this phase of the integrated model, is activation of the EMS cascade. As EMS becomes “normalized” within the community, one would expect increased use of toll-free numbers (for example), to activate the EMS cascade. A way of assessing whether “EMS normalization” has been attained will be through the use of surveys, longitudinally evaluate *changes* in the target population’s perceptions of and attitudes towards prehospital care services; and the number of times the EMS cascade is activated over time. A community that perceives EMS to be approachable, acceptable and “normal” would (increasingly) initiate EMS services.

### The second delay (phase II): reaching care-availability and accommodation, affordability

#### Access concepts

Once emergency care is initiated, the *ability to reach the desired services* is often determined by issues of mobility. The availability (desired services available) and accommodation (ability to reach desired services) and affordability (ability to pay for the services) of emergency transport depends on the physical and geographic infrastructure of the environment (road conditions, traffic rules, season/weather etc.). It is during this second phase of the delay model that EMS competes with taxis and other commercial vehicles that are perceived to be more expeditious and less costly [29, 43].

Though this cost-prohibitive attribute of EMS is not unique to African populations [46], the WHO-led agenda of implementing EC systems across the African region should deeply weigh the effects of a “profitable” EMS system against the number of lives that will be potentially lost if EMS operations were fee-based. For example, recent suggestions of a fee-for-service based model for ambulance use in Ghana, was met with much resistance [47]. Critics cite that even though ambulances are under-utilized, levying a fee for ambulance use will further delay care for the most vulnerable. In the case of Ghana’s National Ambulance Service (NAS), pregnant and laboring women would be at greatest risk if a fee is levied,  since 25%  of all the EMS services provided are obstetrics-related [35].

#### Emergency medicine benchmarks

Measureable outcomes for EC interventions in this phase of the framework should incorporate ambulance response time (the time it takes for ambulances to arrive on-scene), on-scene time (time spent on scene, preparing the laboring woman for transport) and arrival time (duration of time spent transporting the woman to the healthcare facility). Other outcomes could include number of ambulance dispatches and dispatch types.

Country-specific benchmarks should not be based solely on western standards, but rather, contextualized to reflect the geographic and infrastructural barriers specific to the sub-Saharan country and community that the interventions are implemented in. An arrival time of 30 min in Ethiopia, where vehicles yield to emergency vehicles, may not be comparable to Cameroon, where traffic rules do not require yielding to emergency vehicles [40]. Adjustments should be made to standardize these metrics to account for the heterogeneity across communities and countries, before global comparisons (especially to Western standards) are made.

### The third delay (phase III): receiving care-appropriateness

#### Access concepts

The appropriateness of care in the final phase of the framework occurs once care is initiated. *Appropriateness is subjective, and denotes the fit between services rendered, the patient’s needs, and their expectations* [44]. Additionally, * appropriateness is highly dependent on outcome*. In maternal health, survival of both mother and fetus is perhaps the greatest motivator for encouraging laboring mothers to deliver in-hospital, where the staff is expected to be trained to manage labor complications and other obstetric emergencies. Reducing the time to in-hospital interventions is crucial to patient survival, and expeditious services rendered by skilled and well-equipped EC providers can drastically reduce in-hospital maternal mortality.

#### Emergency medicine benchmarks

EC interventions in this phase of the integrated model should take a two-pronged approach. The first approach should focus on bridging the care gap in the pre-hospital context. This would involve training EMS providers-paramedics and Emergency Medical Technicians (EMTs)-to cater to laboring mothers in the field, *before they get to the hospital*. As literature on the “golden hour” shows, expediting urgent care for patients within the first hour after symptom onset, can drastically decrease morbidity and mortality outcomes [48–52]. Having trained EMS providers interact with laboring women *before* they reach the healthcare facility, expedites the woman’s *access to obstetric care*, and consequentially, improves her (and the fetus’) chances of survival.

The second approach to EC interventions in this phase of the model should focus on in-hospital care, with the creation and training of Emergency Medicine providers who are adept at properly triaging patients, and skillfully trained to deliver appropriate life-saving interventions. Deliveries necessitating analgesia, forceps or vacuum extraction, and cesarean sections should be triaged from lower-risk pregnancies with less maternal and/or fetal distress. Emergency and labor wards should be well-equipped and appropriately staffed to deliver the care needed in various emergency situations, so challenging obstetric cases are dealt with appropriately.

As Emergency Medicine takes hold across the continent, and patients get more expedited care, it is hoped that in-hospital maternal mortality and other adverse outcomes will decline. The improvement of such metrics is crucial to patient satisfaction, perceived “appropriateness” of care, and decreased delay in future care-seeking decisions, as shown in Fig. 2.

Sup-par outcomes in the third delay heavily influence the cyclical nature of the entire *Three Delays* model. Thus measurable benchmarks in this phase of the framework should correlate EMS and EM training programs with in-hospital interventions and maternal mortality metrics to ensure that there is a “fit” between the needs and expectations of the target population, and the services rendered by both pre- and in-hospital providers.

## Conclusion

With the advent of the African Federation of Emergency Medicine (AFEM) [54] and the organization of EMS systems across the African continent, it is hoped that there will be sustained efforts to improving *access* to necessary emergency care.

Our proposed conceptual framework integrates maternal health with emergency medicine by juxtaposing the three critical phases of emergency obstetric care with various aspects of healthcare *access*. In each phase EC interventions must be adapted to social and cultural norms, and outcomes tracked longitudinally to evaluate the successes and necessary improvements required each intervention.

**Fig. 3:**
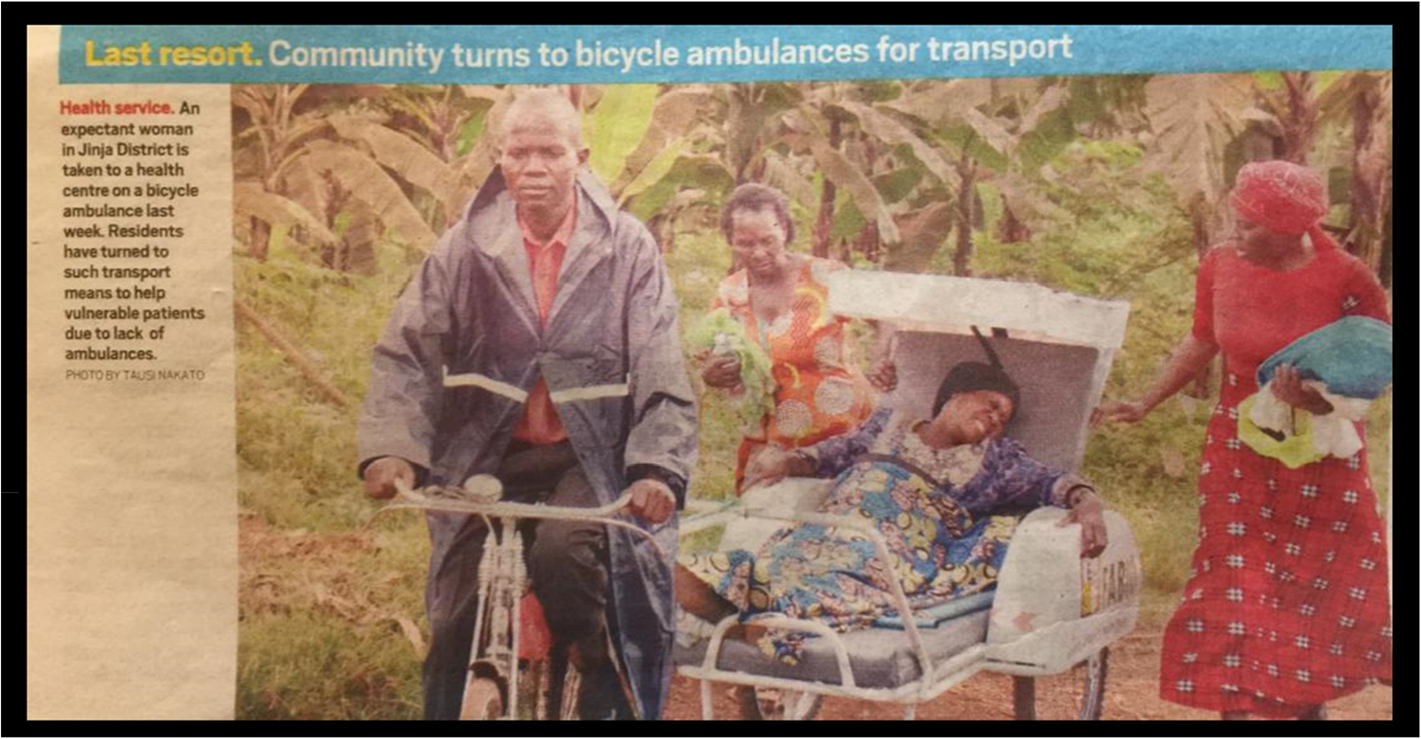
A laboring woman in the Ninja District of Uganda being transported to care by bicycle amubulance operated by Mr. Steven Musoke, a member of the village health team. Legend: Image courtesy of Daily Report Newspaper. Mufumba Isaac (1/27/2019). “When will emergency medical services system be improved?” Available at: https://www.monitor.co.ug/SpecialReports/When-will-emergency-medical-servicessystem-be-improved/688342-4952822-dtrr3k/index.html. Retrieved 3/11/2019. Photo by Tausi Nakato [53]

The adoption of the proposed conceptual framework of access to emergency care will not only make reaching the SDG targets for maternal mortality possible, but also make obsolete, images of laboring women being transported in make-shift ambulances such as those depicted in Fig. 3.

## Data Availability

Not applicable.
